# Lipid Extract From a Vegetable (*Sonchus Oleraceus*) Attenuates Adipogenesis and High Fat Diet-Induced Obesity Associated With AMPK Activation

**DOI:** 10.3389/fnut.2021.624283

**Published:** 2021-04-06

**Authors:** Chih-Yu Chen, Chien-Wen Su, Xiangyong Li, Yinghua Liu, Qian Pan, Tinglan Cao, Jing X. Kang

**Affiliations:** ^1^Laboratory for Lipid Medicine and Technology, Massachusetts General Hospital and Harvard Medical School, Charlestown, MA, United States; ^2^Mucosal Immunology and Biology Research Center, Massachusetts General Hospital and Harvard Medical School, Charlestown, MA, United States; ^3^Institute of Biochemistry and Molecular Biology, Guangdong Medical University, Zhanjiang, China

**Keywords:** obesity, adipogenesis, AMPK, *Sonchus oleraceus*, bitter vegetable

## Abstract

**Scope:** *Sonchus Oleraceus*, named bitter vegetable (BV), has been known to have multiple health benefits such as anti-aging and anti-inflammation. However, the role of BV in the prevention of obesity is unclear. The aim of this study was to examine the effect of BV lipid extracts (BVL) on obesity development.

**Methods and Results:** Following treatments of high fat diet-induced obese mice (C57BL/6J) with BVL (0.3 mg/g of BW per mouse) for a month, mice exhibited a significant reduction in weight gain, blood triglyceride, and fasting blood glucose compared to control mice. Intriguingly, phosphorylated AMPK, a key regulator of nutrient metabolism, was markedly increased in inguinal fat of BVL group. In 3T3-L1 cells, BVL-7 (100 μg/ml), an omega-3 fatty acid-rich fraction from BVL, lowered lipid accumulation, and down-regulated the gene expression of adipocyte markers. The inhibitory effect of BVL occurred at the early stage of adipocyte differentiation, leading to the delay of mitotic clonal expansion. AMPK knockdown by siRNA abolished the inhibitory effect of BVL-7 on adipogenesis, suggesting that AMPK is essential for BVL-regulated adipocyte differentiation.

**Conclusion:** BVL can effectively inhibit adipogenesis through, at least in part, stimulating AMPK pathway and attenuate HFD-induced obesity. Our findings suggest that BVL can be a promising dietary supplement for protection against obesity, and the effective component of BVL can be potentially developed as anti-obesity drugs.

## Introduction

Obesity is mainly developed from energy imbalance, leading to abnormal adipose tissue expansion and subsequently, triggering chronic low-grade inflammation in whole body (1). Therefore, the burden of obesity on public health extends across multiple metabolic disorders such as type 2 diabetes, cardiovascular disease, and cancers (1). Identification of an effective strategy to manage obesity and its related diseases becomes a primary research interest and is important to public health.

Hyperplasia mainly contributes to obesity development by recruiting preadipocytes proliferation and differentiation into mature adipocytes in adipose tissue, a process termed adipogenesis (2). Adipogenesis involves a comprehensive network to form lipid droplets including a cascade of signaling pathway and the stimulation of serial transcriptional factors. So far, both high-fat diet (HFD)-induced obesity mouse model and murine 3T3-L1 cells as an *in vitro* model have been used widely for studying adipogenesis. During the early phase of differentiation, growth-arrested 3T3-L1 preadipocytes reenter the cell cycle, which is characterized by mitotic clonal expansion (MCE) followed by the activation of peroxisome proliferator-activated receptor gamma (PPARγ) and CCAAT enhancer-binding protein α (C/EBPα) (3), subsequently leading to fatty acids synthesis and adipokine production (e.g., leptin and adiponectin). AMP-activated protein kinase (AMPK) acts as a key nutrient sensor on energy metabolism in response to ratios of ATP/ADP (4). There are alternative pathways to produce ATP in non-dividing cells in which activation of AMPK switches anabolic (e.g., synthesis of triglyceride, cholesterol, and glucose) to catabolic metabolism (e.g., fatty acid β-oxidation, glucose uptake, and mitochondria biosynthesis) (5). AMPK, an upstream regulator of PPARγ, acetyl-CoA carboxylase (ACC), and fatty acid synthase (FAS), inhibits lipid accumulation in adipose tissue, and liver once AMPK is phosphorylated (6). AMPK is also modulated by adipokines released from adipocytes including adiponectin and leptin in muscle (7). Accordingly, activated AMPK has been known to reverse insulin resistance, hyperglyceridemia, and body weight gain (8), which are now considered as a therapeutic target of obesity.

Bama, located in the northwest of Guangxi province in China, has been well-known as “longevity village.” Several studies have shown that the elderly in Bama have prolonged lifespan in regarding to their dietary habit and environment (9–11). *Sonchus Oleraceus*, also named bitter vegetable (BV) in Chinese cuisine, is native in Europe and Asia, and commonly found in Bama diets. Its leaves are rich in low molecular weight antioxidants such as polyphenols (12). BV has been known to be an antioxidant, antibacterial (13), anti-inflammatory (14), anti-aging (15), anti-depressant (16), and anti-cancer agent (17). However, the role of BV in obesity development is unknown. To assess the anti-obesity ability of BV, we examined the effects of BV lipid extracts (BVL) on adipogenesis in 3T3-L1 cells and HFD-induced obesity in mice.

## Materials and Methods

All chemicals were purchased from Sigma Aldrich (St. Louis, MO), otherwise specified.

### Animals

Male 8-week-old C57BL/6J mice (Charles River Laboratory, Wilmington, MA) were fed with HFD (D12492, Research Diets Inc., New Brunswick, NJ) containing 60% calories from fat for 3 weeks. Then, the mice were randomly distributed to 2 groups: HFD group and HFD+BVL group for another 4 weeks. The body weight and food intake were recorded weekly. In the end of the experiment, mice were fasted for 12 h and then euthanized to harvest adipose tissues and blood for biochemical analysis. The Subcommittee on Research Animal Care (SRAC) serves as the Institutional Animal Care and Use Committee (IACUC) for Massachusetts General Hospital (MGH), reviewed and approved all experimental procedures detailed in this study (Animal protocol No: 2010N000038).

### Dosage Information

During a 4-week dietary intervention, HFD, and HFD+BVL groups were oral-gavaged daily by 100 μl of olive oil and 100 μl of olive oil containing 10 mg BVL (0.3 mg/g of BW per mouse), respectively. The design of BVL dosages was based on pre-experiments and our previous work, at which no toxicity observed to mice (15). The dose of 10 mg/30 g of mouse per day was equivalent to human consumption of BVL of 27 mg/kg, which equates to a 1.6 g dose of BVL for a 60 kg person according to the formula for dose translation based on surface area (18).

### BV Lipid Extraction

Dried plant of BV was subjected to lipid extraction using Bligh and Dyer's method (19) and lipid separation was performed by thin layer chromatography methodology (TLC). Briefly, the entire BV plant was grinded into powder and then mixed under chloroform and methanol (2:1, v/v) for overnight extraction at 4°C. The mixture was added with distilled water and processed to centrifuge at 3000 rpm for 5 min. The chloroform layer containing lipid was collected and dried under nitrogen. The BVL was weighed and suspended in olive oil for the animal experiment. For the cell culture experiment, BVL was further purified by two-step solvent system on silica gel TLC plate. In the first step, the mobile phase was consisted of Heptane: Diethyl Ether: Acetic Acid (60:40:3, v/v/v), and the bottom layer rich in phospholipid was collected (Supplementary Figure 1A, arrow). The phospholipid fraction obtained from TLC plate was further separated using mobile phase containing Chloroform: Methanol: Acetic acid (100:30:3, v/v/v) in the second step. The front layer of TLC plate which was collected and extracted was named as BVL-7 (Supplementary Figure 1B, arrowhead) which were later reconstituted in ethanol, stored at −20°C, and ready for *in vitro* experiments.

### Fatty Acid Analysis

BVL or BVL-7 was methylated by adding 1:1 hexane and 14% boron trifluoride/methanol and heated at 100°C for an hour. Fatty acids methyl esters were analyzed by a fully automated 6890N Network Gas Chromatography equipped with a flame-ionization detector (Agilent Technologies, Palo Alto, CA). Individual fatty acid was determined by retention time using a reference standard, GLC461 (Nu-Chek Prep, Elysian, MN).

### Body Composition

Body composition including lean tissue, fat, and body fluid was measured using Bruker Minispec Live Mice analyzer (Bruker Optics Inc., Billerica, MA) and the method was previously described (20).

### Biochemical Analysis

Blood was collected and processed to centrifuge at 3000 rpm for 10 min to obtain plasma. Triglyceride (TG) was measured by the Center of Comparative Medicine in MGH. Insulin was determined by ultra-sensitive mouse insulin ELISA (Crystal Chem Inc., Grove, IL). Prior to sacrifice, blood glucose level was measured by glucometer (Bayer HealthCare LLC, Tarrytown, NY).

### Immunohistology

The inguinal and epididymal adipose tissues were collected and fixed in 4% paraformaldehyde-PBS. The paraffin embedding and hematoxylin and eosin (H&E) staining were performed by pathology core in MGH. The slices were observed using ECLIPSE E600 microscope (Micro Video Instruments Inc., Avon, MA) at 400X magnification. The adipocyte size was measured using Case viewer (Servicebio Inc, Woburn, MA).

### Cell Culture

3T3-L1 mouse fibroblasts (a gift from Dr. Chongzhao Ran in MGH) were maintained in 10% FCS-DMEM. Preadipocytes were differentiated by 10% FBS-DMEM with adipogenic DMI cocktail (1 μg/ml insulin, 0.5 mM 1-isobutylmethylxanthine and 1 μM Dexamethasone) at 2-day post-confluence, referred as to day 0 (D0). The medium was changed to 10% FBS-DMEM with 1 μg/ml insulin at D2 and replaced with 10% FBS-DMEM every other day until D8. 100 μg/ml of indicated BVL fractions were added to medium throughout the differentiation. The lipid droplets in adipocytes were stained with Oil Red O (ORO) and photographed. ORO dye was extracted by isopropanol and measured at the wavelength of 490 nm using Epoch microplate spectrophotometer (Biotek, Winooski, VT). FCS, FBS, and DMEM were purchased from Gibco (ThermoFisher Scientific, Grand Island, NY).

### Immunoblotting

Total proteins from cells/tissues were extracted using RIPA buffer supplemented with protease inhibitor cocktail and phosphatase inhibitors at indicated time points. Samples were subjected to SDS-polyacrylamide gel electrophoresis and transferred to methanol-pretreated PVDF membranes (Millipore Corp., Bedford, MA, USA). Primary antibodies used in this study included p-AMPK (Thr(P)-172), total AMPK (Genetex, Irvine, CA), FAS, p-PPARγ, p-C/EBPα, AP2, p-C/EBPβ, CDK2, and Cyclin A (Cell signaling, Beverly, MA). The loading control: β-actin and secondary-HRP-conjugated mouse as well as rabbit antibodies were obtained from Santa Cruz Biotechnology (Dallas, TX). Protein bands were detected with SuperSignal West Pico Chemiluminescent Substrate (ThermoFisher Scientific, Grand Island, NY) by autoradiography.

### PCR Analysis

Total mRNAs from cells/tissues were extracted with TRIzol^®^ (Invitrogen, Grand Island, NY). The cDNA was synthesized by iScriptTM system (Bio-Rad, Hercules, CA) and reverse transcription reaction was performed using PTC-100 programmable thermal controller (MJ Research Inc., Waltham, MA). A real time PCR was performed using an iTaqTM universal STBR^®^ green Supermix (Bio-Rad, Hercules, CA) in an Mx3005P qPCR thermocycler (Agilent Technologies, Santa Clara, CA). All values were normalized by β-actin expression and further analyzed using the ΔΔCT method. The sequences of primers used in semi-quantitative PCR were listed as following, PPARγ (forward, 5′-CCC AAT GGT TGC TGA TTA CAA AT-3′, and reverse, 5′-CTA CTT TGA TCG CAC TTT GGT ATT CT-3′); C/EBPα (forward, 5′-GGT TTA GGG ATG TTT GGG TTT TT-3′, and reverse, 5′-AAG CCC ACT TCA TTT CAT TGG T-3′); C/EBPβ (forward, 5′-AGC GGC TGC AGA AGA AGG T-3′, and reverse, 5′-GGC AGC TGC TTG AAC AAG TTC-3′); FAS (forward, 5′-GCC ACC CAC CGT CAG AAG-3′, and reverse, 5′-TGT CAC ATC AGC CAC TTG AGT GT-3′); AdipoQ (forward, 5′-GAT GCA GGT CTT CTTG GTC CTA A-3′, and reverse, 5′-GGC CCT TCA GCT CCT GTC A-3′); IL-6 (forward, 5′-TCG GAG GCT TAA TTA CAC ATG TTC-3′, and reverse, 5′-TGC CAT TGC ACA ACT CTT TTC T-3′); Leptin (forward, 5′-CAC ACA CGC AGT CGG TAT CC-3′, and reverse, 5′-AGC CCA GGA ATG AAG TCC AA-3′); MCP-1 (forward, 5′-GCT TGA GGT GGT TGT GGA AAA-3′, and reverse, 5′-CTC ACC TGC TGC TAC TCA TTC-3′); β-actin (forward, 5′-AGA TGA CCC AGA TCA TGT TTG AGA-3′, and reverse, 5′-CAC AGC CTG GAT GGC TAC GT-3′).

### AMPK Knockdown

To knockdown AMPK expression in 3T3-L1 cells, 100% confluent preadipocytes were transfected with AMPK^α1/2^ siRNA (Santa Cruz Biotechnology, Dallas, TX) together with lipofectamin RNAiMax (Invitrogen, Grand Island, NY). In a 12-well plate scale, 20 pmol siRNA and 4 μl of lipofectamine were diluted in 100 μl Opti-MEM (ThermoFisher Scientific, Grand Island, NY), respectively, and then siRNA-Lipo mixture were incubated for 20 min at room temperature. The medium was replaced by DMI medium with/without BVL-7 and the effectiveness of AMPK knockdown was determined on adipocytes at D8 by immunoblotting.

### Flow Cytometry

Two-day post-confluent 3T3-L1 cells were incubated in DMI adipogenic induction medium with/without BVL-7 for 16 and 24 h. The cells pellets were harvested at each time points and fixed with 70% ice-cold ethanol at −20°C overnight. The cells were incubated with 0.5 mg/ml RNase for 30 min at 37°C and stained with 10 μg/ml propidium iodine solution prior to cell cycle analysis using LSR II flow cytometer (BD Biosciences, Franklin Lakes, NJ).

### Statistical Analysis

All values were presented as mean ± SEM. The differences between two groups were evaluated by two-tail *T*-test using GraphPad Prism software. The data was subjected to one-way ANOVA within three groups, followed by TukeyHSD multiple comparison test and least significance difference test (LSD test). The differences in means were considered statistical significance at *p* < 0.05.

## Results

### BVL Attenuates HFD-Induced Obesity Development in Mice

**Figure 1:**
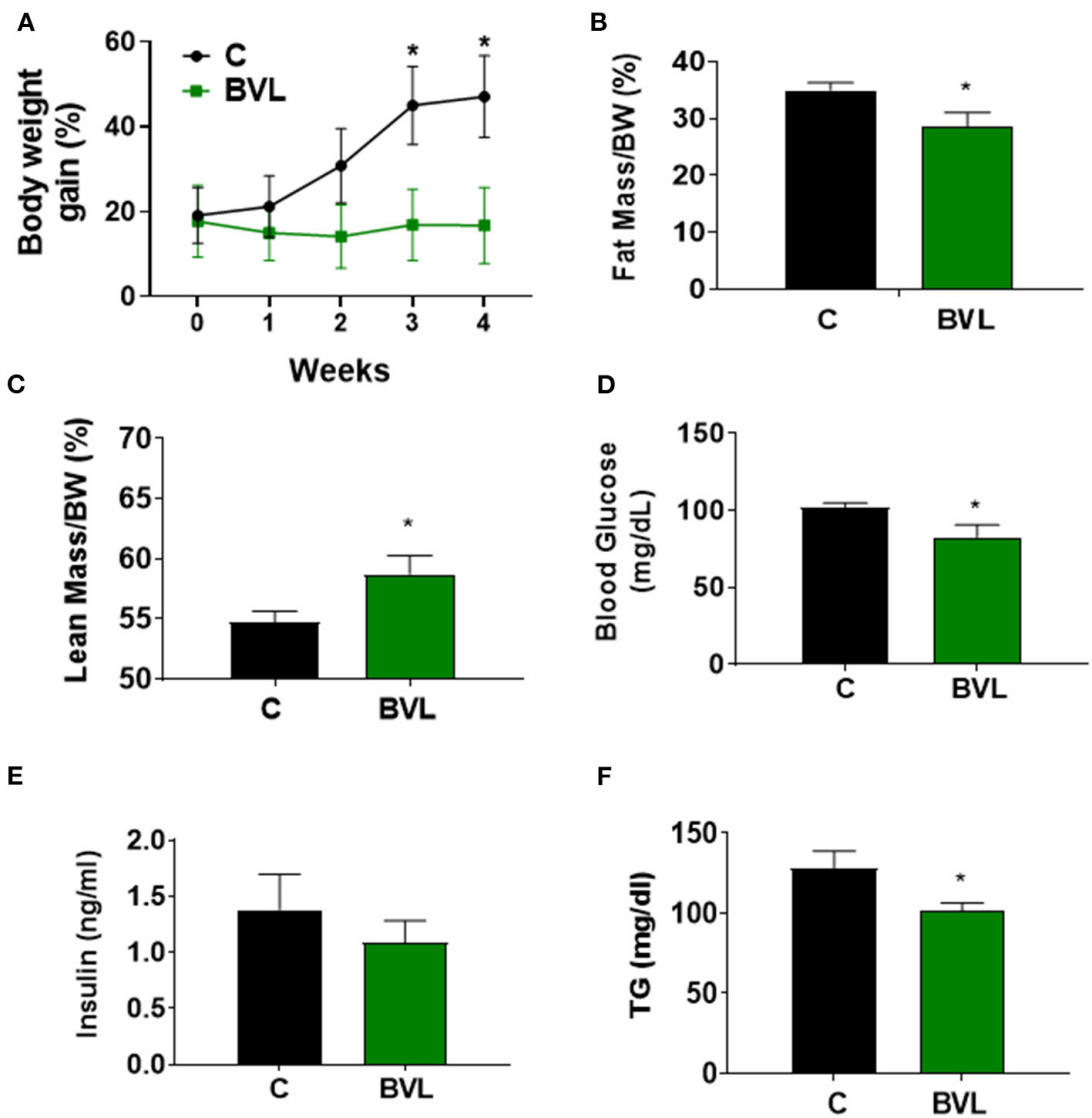
BVL reduces body weight gain in HFD-induced obese mice. After a 3-week HFD feeding, animals were randomly assigned to two groups, HFD and HFD+BVL, which were fed HFD in the absence and presence of BVL for another 4 weeks. **(A)** The changes of body weight gain. The result was shown by combing two independent experiments. w/wo: with/without. Body composition was measured and the percentage of fat mass **(B)** as well as lean mass **(C)** was shown. Fasting blood glucose **(D)**, insulin levels **(E)**, and plasma TG levels **(F)** were compared between groups. The values were presented using mean ± SEM (*n* = 4–6). *p < 0.05.

To investigate the effect of BVL on obesity development, male C57BL/6J mice were fed HFD for 7 weeks and supplemented daily with BVL dissolved in 100 μl olive oil (10 mg/30 g of mouse) or olive oil alone as control for the last 4 weeks. The total food intake during the experiment was nearly equivalent between groups (Supplementary Figure 2A). The body weight gain in BVL group was significantly reduced after 2 weeks of supplementation relative to control (C) group (*p* < 0.05) (Figure 1A). The analysis of body composition indicated a decrease of fat mass and an increase of lean mass in BVL group (Figures 1B,C). Consistently, the masses of inguinal (Ing) fat and epididymal (Epi) fat in BVL group were significantly less than C group while muscle mass in BVL group was higher than C group (*p* < 0.05, Supplementary Table 1). Moreover, we found that the fasting blood glucose, plasma insulin level, and plasma TG level were reduced in BVL group relative to control group (Figures 1D–F). Our results demonstrate that BVL is able to reduce HFD-induced body weight gain and improved metabolic parameters. In contrast, BV water hot extracts did not impact any body weight loss, and the trend of weight gain was similar to C group (Data was not shown here.). This indicates that the lipid component in BV plays a key role on obesity development.

### BVL Inhibits Fat Accumulation and Inflammation in Adipose Tissue

**Figure 2:**
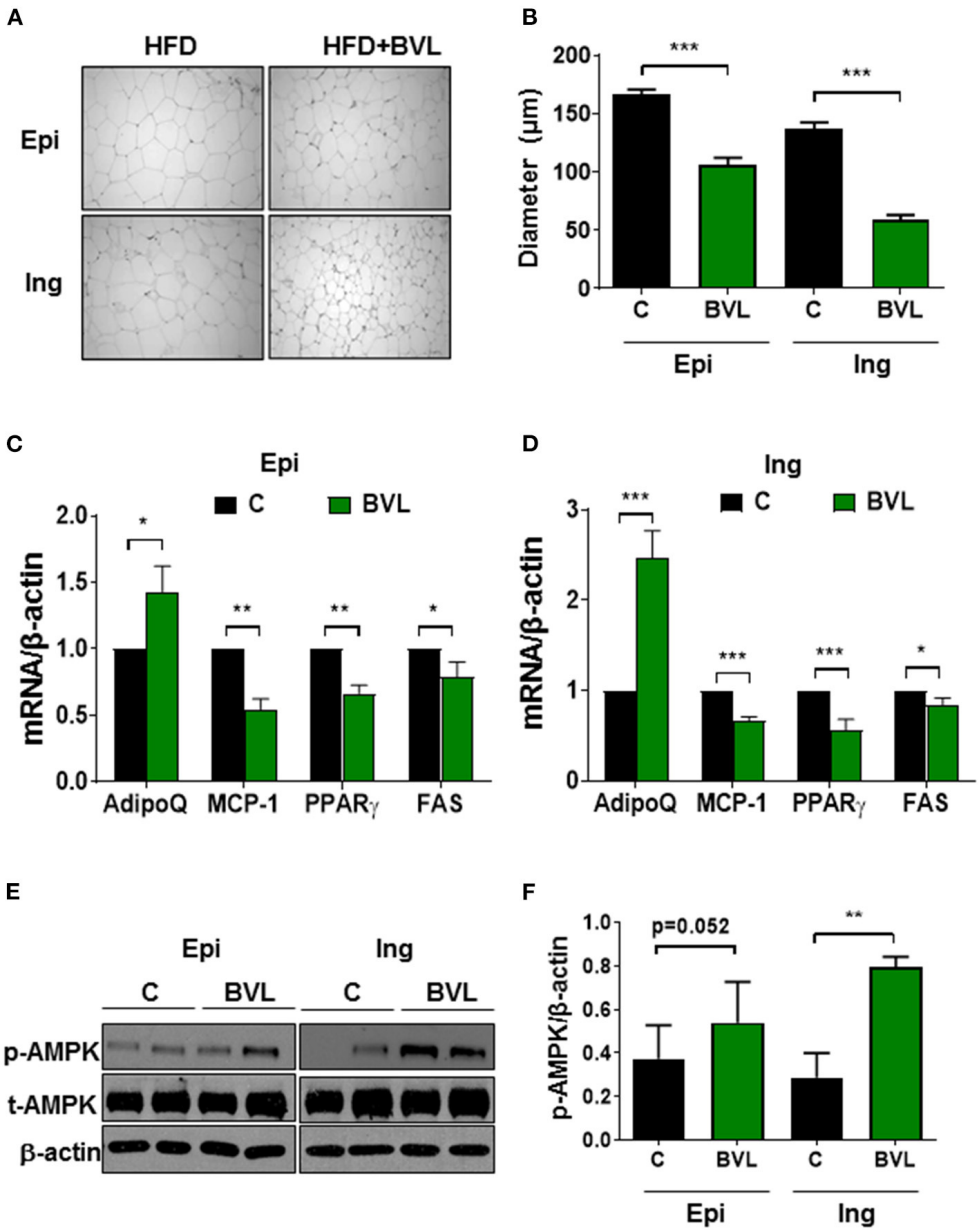
BVL attenuates adipocyte expansion and inflammation in adipose tissue. **(A)** The adipocyte size in epididymal (Epi) and inguinal (Ing) fat pad was shown in this representative image and the quantification of adipocyte size was shown in **(B)**. The gene expressions in Epi **(C)** and Ing **(D)** were performed using qPCR. The representative image of immunoblotting **(E)** and quantification of p-AMPK expression **(F)** in Ing and Epi were measured using software Image J. The values were presented using mean ± SEM (*n* = 3–4). The experiment was repeated twice with similar results and to avoid the huge variation, the data shown here was from the second experiment. *p < 0.05, **p < 0.01, ***p < 0.001.

HE staining of adipose tissue revealed a significant difference in adipocyte size between groups (Figures 2A,B). The smaller adipocyte size was observed in BVL group, suggesting an inhibitory effect of BVL on fat accumulation in Ing and Epi fat. Accordingly, BVL-fed mice had significantly lower mRNA expressions of adipogenic genes, PPARγ, and FAS relative to control mice. In addition, we analyzed inflammation-related molecules of adipose tissue and found that BVL-fed mice had a significantly lower mRNA expression of MCP-1, and a higher mRNA level of AdipoQ relative to control group (Figures 2C,D). Intriguingly, BVL-treated mice exhibited an increase in AMPK phosphorylation (p-AMPK) in Ing adipose tissue compared to control mice (Figures 2E,F). These results suggest that BVL suppresses body weight gain by decreasing fat accumulation and inflammation-related gene expression.

### BVL-7, an Omega-3 Fatty Acid-Rich Fraction of BVL, Suppresses Adipogenesis, and Its-Related Transcriptional Factors in 3T3-L1 Cells

**Figure 3:**
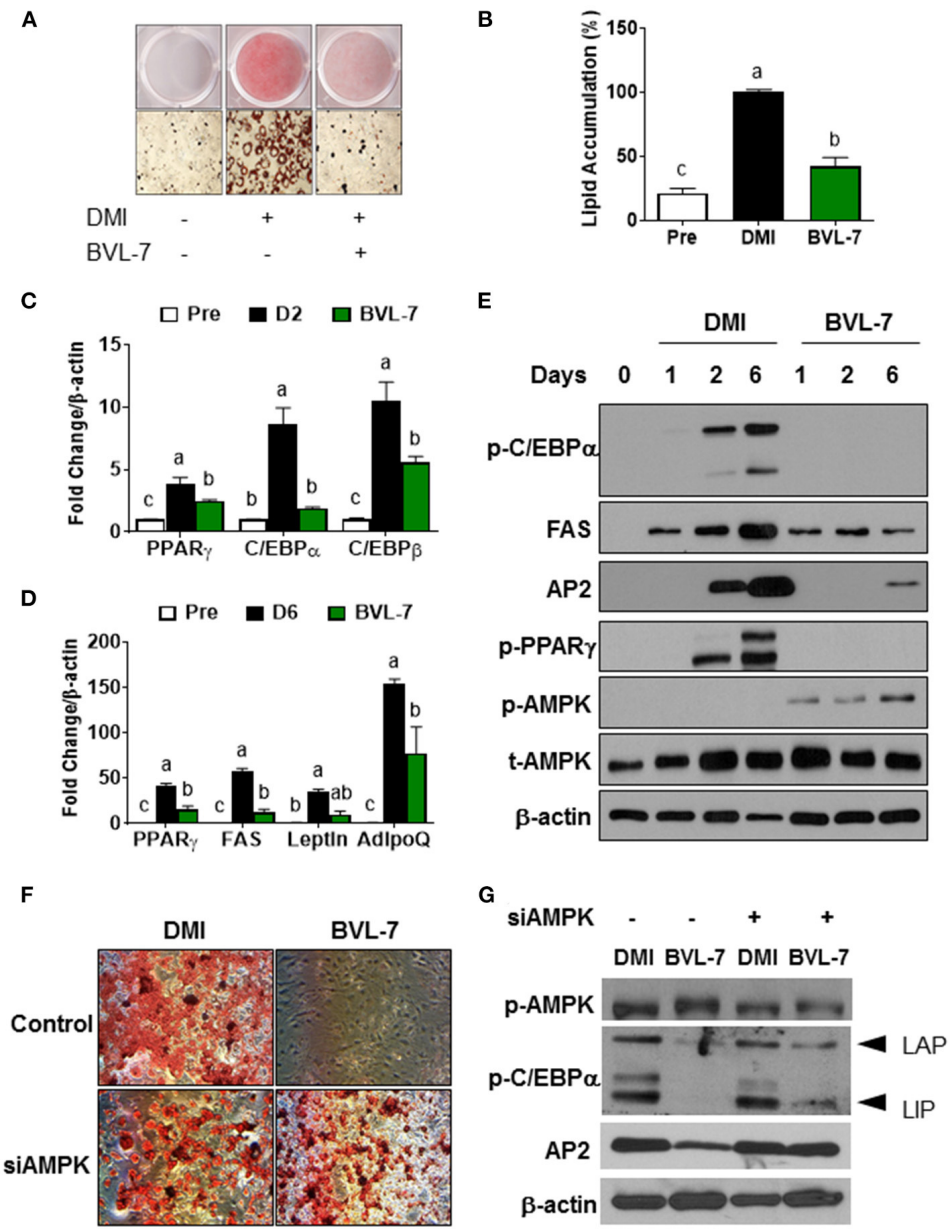
BVL-7 inhibits adipocyte differentiation through AMPK activation. 3T3-L1 preadipocytes were differentiated with or without BVL (100 μg/ml) at the indicated time. **(A,B)** The representative image and quantitative analysis of lipid accumulation from adipocytes on D6 were shown using ORO staining. **(C,D)** The mRNA expressions of adipocyte markers were measured on the early and late stages of differentiation, indicating D2 and D6, respectively. **(E)** The protein samples were collected on the indicated time points such as D1, D2, and D6. The representative image was shown. siAMPK transfection was performed in 90% confluent cells 24 h before differentiation and then, the cells were differentiated to adipocytes with/without BVL. The lipid accumulation was shown using ORO staining **(F)** and adipogenesis-related markers as well as p-AMPK were measured by immunoblotting **(G)**. The values were presented using mean ± SEM and each experiment was repeated at least 3 times. Different letters were presented as statistical difference (*p* < 0.05).

To identify the effective fraction of BVL on adipogenesis, we utilized TLC method to separate the crude lipid extracts into several fractions (Supplementary Figure 1C) and tested for their effects on adipogenesis. We found that a fraction (BVL-7) had a mostly effective inhibition on adipogenesis while other fractions (BVL-1-6) had little effect on adipocyte differentiation (Supplementary Figure 2A). GC analysis showed that BVL-7 was composed of ALA up to 72% and LA content down to 3.6%, with a very low ratio of omega-6/omega-3 about 0.05 (Supplementary Figure 1C). Subsequently, we used this BVL-7 fraction to test for its effect on adipogenesis throughout the experiment. Differentiation of 3T3-L1 preadipocytes to adipocytes was induced with DMI containing different concentrations of BVL-7 (0-200 μg/ml), and we found that 100 μg/ml of BVL-7 was effective in inhibiting adipogenesis without any cell toxicity (Supplementary Figures 2B–D). After 6 days of differentiation, ORO staining revealed that lipid droplets accumulated in BVL-7-treated cells were markedly reduced by approximately 75% relative to DMI-treated control adipocytes (Figures 3A,B). Accordingly, mRNA expression of early adipogenic transcription factors such as PPARγ, C/EBPα, and C/EBPβ, were suppressed in BVL-7-treated cells relative to DMI control group on Day 2 (Figure 3C). In the terminal stage of adipogenesis (Day 6), mRNA expression of fatty acid synthase (FAS), Leptin, and adiponectin (AdipoQ) in BVL-7-treated cells were down-regulated relative to DMI control group (Figure 3D). In consistent with our PCR data, the protein levels of p-PPARγ, p-C/EBPα, FAS, and AP2 during adipocyte differentiation were reduced by BVL-7 (Figure 3E). These results demonstrate that BVL-7 could effectively suppress adipogenesis-related gene expression and the formation of adipocytes.

### BVL-7 Blocks Adipogenesis Partially Through AMPK Activation

In consistent with the observation that the level of phosphorylated AMPK was increased in Ing adipose tissue of BVL-treated mice, we found that BVL-7 dramatically increased AMPK phosphorylation during the differentiation of 3T3-L1 cells to adipocytes (Figure 3E). To examine whether AMPK mediates the inhibitory effect of BVL-7 on adipogenesis, we employed siRNA to knockdown AMPK expression in 3T3-L1 cells and then examined the effect of BVL-7 in these cells. We found that AMPK knockdown abolished the inhibitory effect of BVL-7 on adipogenesis as evidenced by restored lipid accumulation and protein levels of AP2 and p-C/EBPα (Figures 3F,G). These data suggest that AMPK is essential for the inhibitory effect of BVL-7 on adipocyte differentiation.

### BVL-7 Inhibits Mitotic Cell Expansion During Adipocyte Differentiation

**Figure 4:**
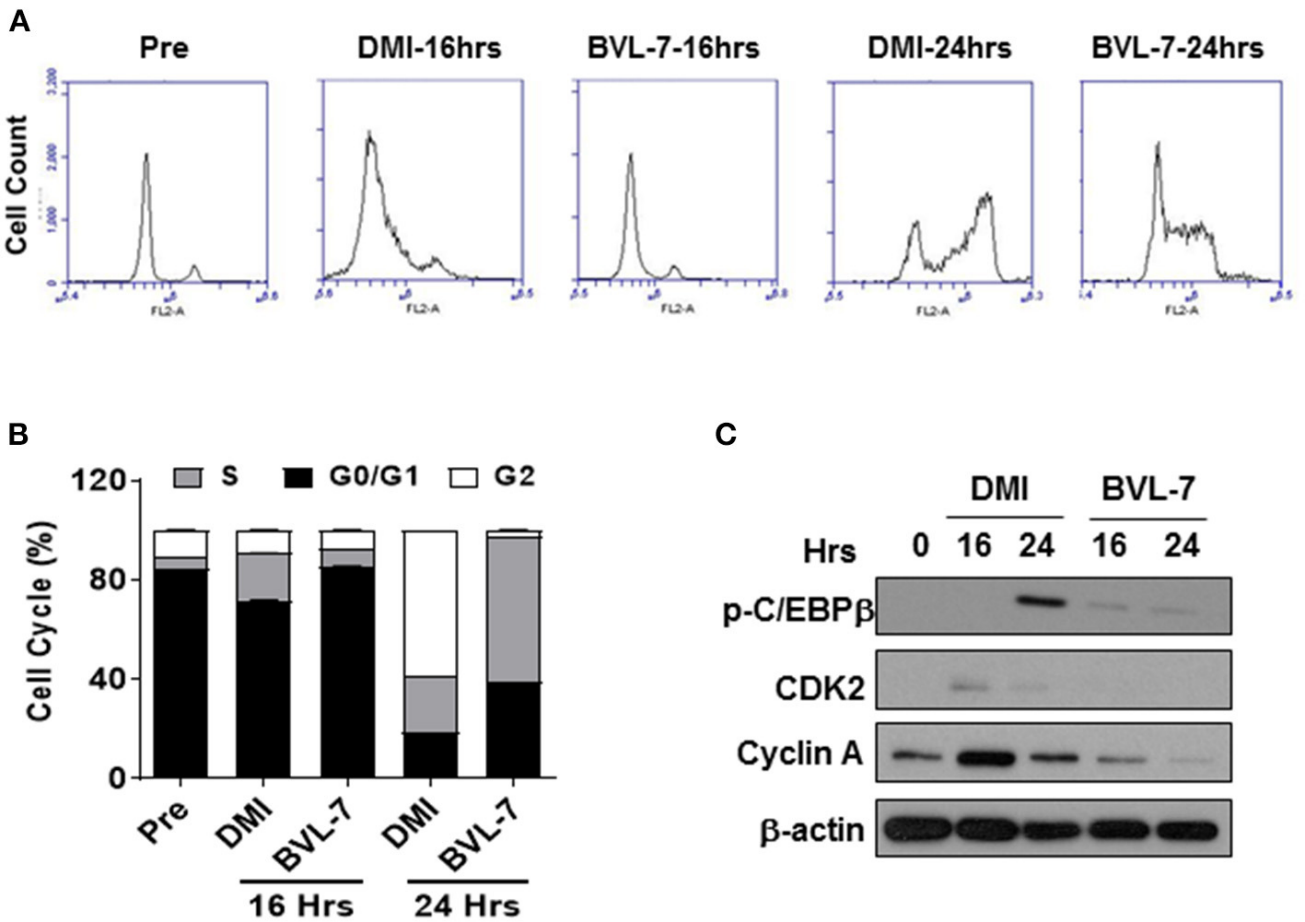
BVL-7 suppresses mitotic clonal expansion in the early stage of differentiation. **(A)** The cell cycle analysis was performed by flow cytometry and **(B)** the distribution of each phase in the cell cycle was shown. **(C)** The protein levels of cell cycle regulators were measured, and the representative image was shown. The values were presented using mean ± SEM (*n* = 3).

As BVL-7 was shown to suppress the expression of C/EBPβ and elevate the level of phosphorylated AMPK at the early stage of adipocyte differentiation (Figure 3E), we next examined whether BVL-7 has any effect on MCE program of differentiating 3T3-L1 cells by flow cytometry. Within 16- and 24-h post-induction of adipocyte differentiation, 3T3-L1 cells treated with 100 μg/ml BVL-7 clearly exhibited a delayed entry into S phase and its subsequent transition into G2/M phase relative to DMI control cells (Figures 4A,B). Consistently, cell cycle regulators, cyclin A and CDK2, which are responsible for G1/S transition, were inhibited by BVL-7 relative to DMI control cells. The protein level of p-C/EBPβ was reduced by BVL-7 compared to control at 24 h of post-differentiation (Figure 4C). These data suggest that BVL-7 can suppress MCE process, which may contribute to reduced adipocyte differentiation.

## Discussion

**Figure 5:**
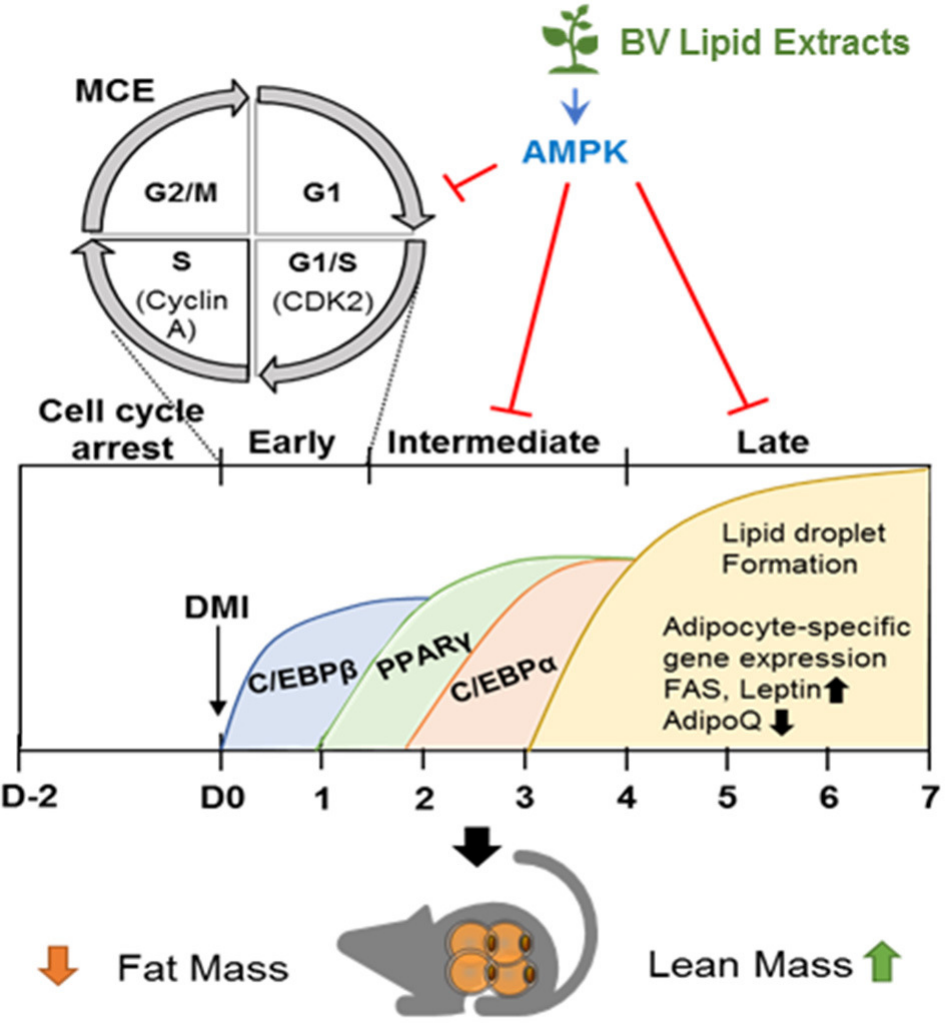
BV lipid extracts inhibit adipogenesis through regulating AMPK. The diagram illustrates a proposed mechanism by which BV lipid extracts limit adipogenesis including MCE suppression, decreased expression of transcriptional factors, and of adipocyte markers through, at least in part, stimulating AMPK. This inhibitory effect of BV lipid extracts are associated with the loss of fat mass and an increase of lean mass.

Understanding the complexity of adipogenesis is important for the management of human chronic diseases, as adipocyte dysfunction largely contributes to obesity and its related metabolic syndromes (21). Suppressing adipogenesis is considered as a therapeutic approach to combat obesity (22). Dietary modification is one of global strategies for long-term weight loss. The identification of safe, natural ingredients for targeting adipogenesis is urgently need (23). Herein, we have demonstrated that treatment of HFD-induced obese mice with BVL for 1 month is capable of significantly reducing body weight gain with decreases of fat accumulation, TG level, and adipogenic markers without the difference in food intake. In 3T3-L1 cells, we found that BVL-7 inhibits MCE, down-regulates transcriptional factors that are required for initiating adipogenesis including C/EBPβ, C/EBPα, and PPARγ, and suppresses lipid synthesis-related factors at the terminal stage of adipocyte differentiation such as FAS, AP2, and leptin (Figure 5). The inhibitory role of BV lipid extracts on weight gain and adipogenesis was associated with an increase of p-AMPK expression and AMPK-relevant anti-adipogenic events.

Cell proliferation is energetically demanding, and AMPK acts as an energy sensor restraining cell growth when energy reserves are insufficient (24). AMPK also plays an important role in the determination of mesenchymal stem cell differentiation where AMPK favors differentiating toward to osteoblasts instead of adipocytes when calorie restriction (25). In our study, BVL-7 induced AMPK phosphorylation after 24 h of DMI-induced adipocyte differentiation, a concomitant with sustaining G1/S cell cycle arrest and blocking MCE progression, suggesting that BVL-7 triggers AMPK phosphorylation at the early stage of differentiation and alters the cell cycle of differentiating preadipocytes.

AMPK, a central energy-sensing enzyme involved in glucose and lipid metabolism, has been considered a therapeutic target for metabolic diseases and cancers (26). Research in hepatic cells demonstrated that AMPK is an upstream target for improving glucose/insulin resistance by regulating Akt/GSK-3β (27) and Akt/PI3K (28). Studies in adipocytes showed that AICAR (5-aminoimidazole-4-carboxamide ribonucleotide), an analog of AMP to stimulate AMPK, inhibits adipogenesis and increases phosphorylation of ACC, a key regulator of fatty acid synthesis and oxidation (29, 30). Moreover, adipocytes isolated from AMPKα1^−/−^ mice exhibited increased lipogenesis in adipose explants (31) and in liver, phosphorylated AMPK was reported to decrease lipogenesis transcription factor, SREBP1c (sterol regulatory element-binding protein 1c) as well as its downstream molecules including ACC, FAS, and SCD-1 (stearoyl Co-A desaturase-1) (32, 33). Additionally, our previous data showed that mice treated with BV-contained diet have less hepatic steatosis and elevated AMPK expression in liver relative to control (15). In consistent with our findings, BVL-7 activated p-AMPK with a decrease of FAS and lipid accumulation in 3T3-L1 cells, and the anti-adipogenic effect of BVL-7 was abolished in AMPK knockdown cells. In a HFD-induced obese mouse model, relative to control group, we observed that BVL-treated mice are resistant to weight gain associated with activated AMPK phosphorylation.

As literature has shown that white adipocytes mainly act as fat storage organ, brown adipocytes play a differential role that function for energy expenditure due to high mitochondria content and characteristic UCP-1 (uncoupling binding protein-1) expression. AMPK can be stimulated by cold exposure and adrenergic nerves, and further activates brown adipocyte or beige cell differentiation *in vitro* as well as thermogenesis *in vivo* (34). Interestingly, we also found that BVL-treated mice have higher rectal temperature than control group when they are exposed to 4°C (Supplementary Figure 3B) as a result of BVL-induced AMPK and indirect influence on thermogenesis. In addition to our findings, other possibilities for BVL contributing to the anti-obesity effect such as thermogenesis cannot be excluded; however, more studies are necessary to confirm the effect of BVL on energy metabolism. In brain, AMPK is highly expressed in hypothalamus and has been emerged as a master sensor of appetite. Fasting or adenovirus-activated AMPK in hypothalamic regions promotes AMPK activity and further leads to an increase of food intake and weight gain (35, 36). Nevertheless, mice with global knockout of AMPK have weight gain and more adiposity compared to WT mice (37). In our present study, similar food intake between BVL and control groups was observed; this might be due to BVL-regulated AMPK function differently in terms of organ plasticity, but more research is needed. Overall, BV lipid extracts alters the adipogenesis and weight gain associated with AMPK phosphorylation.

MCE is one of the earliest events of adipogenesis that growth-arrested preadipocytes synchronously re-enter the cell cycle and undergo one/two rounds of cell divisions (38). C/EBPβ is an essential transcription factor to initiate MCE and other transcriptional activation of KLF5 (Kruppel Like Factor 5), PPARγ, and C/EBPα (38, 39). Our data showed that the anti-adipogenic role of BVL-7 is through the inhibition of MCE at early stage of adipocyte differentiation (Figure 4) and constitutively, of adipogenic transcription factors as well as lipid droplet formation (Figures 3C–E). Moreover, adipocytes incubated with BVL-7 during days 0–4 or days 0–6 exhibited more than 50% reduction in intracellular lipid content compared with control adipocytes while adipocytes treated with BVL during days 0–2, days 2–4, or days 4–6 had similar levels of lipid accumulation as control adipocytes (Supplementary Figure 2E). Additionally, Sirt6 (NAD-dependent protein deacetylase sirtuin-6) could activate AMPK and is essential for controlling MCE (40). Although further investigation for the interaction between BVL-7 and Sirt6 during adipogenesis is needed, we found that p-AMPK is activated after 1 day of BVL-7 treatment and continued to the end of adipocyte differentiation (Figure 3E). Our results indicate that BVL-7 possibly suppresses the early events of adipogenesis, rather than inhibits lipid droplet formation at the terminal stage of adipocyte differentiation.

BV (*Sonchus Oleraceus*) has been found as an anti-inflammatory agent in various chronic inflammation models. In aged mice, our group previously demonstrated that BV alleviates aging-induced inflammation (15). BV has been reported to suppress pro-inflammatory cytokines including TNFα (tumor necrosis factor α), and IL-6 (interlukin-6), inhibit their related mediators such as TLR4 (toll like receptor 4) and NF-κB, and reduce leukocyte recruitment upon LPS stimulation (14, 41). Accordingly, our results showed that BVL decrease MCP-1 gene expression in adipose tissue in a HFD-induced obesity model, as chronic low-grade inflammation is accompanied with white adipose tissue expansion and MCP-1 is important chemokine by promoting macrophage infiltration in fat tissue (42). Moreover, current human and animal studies have reported that adipoQ is anti-inflammatory factor and the adipoQ level is positively correlated to lower BMI, and insulin sensitivity (43). Here, BVL-treated mice showed the elevated level of adipoQ in adipose tissue relative to control mice. These results have demonstrated that BVL is able to modulate inflammation in obese condition.

In summary, our findings present evidence for a protective effect of BV lipid extracts against obesity development in both cellular and animal levels. The observed beneficial effects of BV lipid extracts are likely through activation of AMPK, which leads to the delay of mitotic clonal expansion, down-regulation of adipogenic genes including C/EBPα, PPARγ, and FAS, and subsequent reduction of adipocyte differentiation and lipid accumulation *in vitro*. The protective effect of BV lipid extracts on HFD-induced obesity is illustrated in our study (Figure 5). Although it is possible that omega-3 fatty acid of BV lipid extracts contributes to the anti-obesity effect of BVL or BVL-7 and identification of specific components responsible for the anti-obesity effect remains for future study, our findings have implications for the utility of the vegetable as a safe and effective agent for the prevention of obesity.

## Data Availability Statement

The raw data supporting the conclusions of this article will be made available by the authors, without undue reservation.

## Ethics Statement

The animal study was reviewed and approved by Institutional Animal Care and Use Committee for Massachusetts General Hospital.

## Author Contributions

C-YC and JXK, conceptualization and methodology. C-YC, C-WS, XL, YL, QP, and TC, validation and investigation. C-YC, formal analysis, writing—original draft preparation, visualization, and project administration. JXK, resources, writing—review, editing, supervision, and funding acquisition. All authors have read and agreed to the published version of the manuscript.

## Conflict of Interest

The authors declare that the research was conducted in the absence of any commercial or financial relationships that could be construed as a potential conflict of interest.

## References

[1] Chawla A Nguyen KD Goh YP. Macrophage-mediated inflammation in metabolic disease. Nat Rev Immunol. (2011) 11:738–49. 10.1038/nri3071 21984069 PMC3383854

[2] Shepherd PR Gnudi L Tozzo E Yang H Leach F Kahn BB. Adipose cell hyperplasia and enhanced glucose disposal in transgenic mice overexpressing GLUT4 selectively in adipose tissue. J Biol Chem. (1993) 268:22243–6. 10.1016/S0021-9258(18)41516-5 8226728

[3] Rosen ED MacDougald OA. Adipocyte differentiation from the inside out. Nat Rev Mol Cell Biol. (2006) 7:885–96. 10.1038/nrm2066 17139329

[4] Hardie DG. The AMP-activated protein kinase pathway–new players upstream and downstream. J Cell Sci. (2004) 117(Pt. 23):5479–87. 10.1242/jcs.01540 15509864

[5] Hardie DG. AMPK: a key regulator of energy balance in the single cell and the whole organism. Int J Obes. (2008) 32(Suppl. 4):S7–12. 10.1038/ijo.2008.116 18719601

[6] Srivastava RAK Pinkosky SL Filippov S Hanselman JC Cramer CT Newton RS. AMP-activated protein kinase: an emerging drug target to regulate imbalances in lipid and carbohydrate metabolism to treat cardio-metabolic diseases: thematic review series: new lipid and lipoprotein targets for the treatment of cardiometabolic diseases. J Lipid Res. (2012) 53:2490–514. 10.1194/jlr.R025882 22798688 PMC3494254

[7] Stern JH Rutkowski JM Scherer PE. Adiponectin, leptin, and fatty acids in the maintenance of metabolic homeostasis through adipose tissue crosstalk. Cell Metab. (2016) 23:770–84. 10.1016/j.cmet.2016.04.011 27166942 PMC4864949

[8] Ruderman NB Carling D Prentki M Cacicedo JM. AMPK, insulin resistance, and the metabolic syndrome. J Clin Invest. (2013) 123:2764–72. 10.1172/JCI67227 23863634 PMC3696539

[9] Yang X Wang X Yao H Deng J Jiang Q Guo Y. Mitochondrial DNA polymorphisms are associated with the longevity in the Guangxi Bama population of China. Mol Biol Rep. (2012) 39:9123–31. 10.1007/s11033-012-1784-8 22729909

[10] Tokui N Minari Y Kusunoki K Yoshimura T Yamamoto T Minagawa M. Evaluation of dietary intake using carbon and nitrogen isotope analysis of human hair of Chinese living in southern part of China. J UOEH. (2000) 22:219–28. 10.7888/juoeh.22.219 11019388

[11] Yang J. On the Bama longevity zone and the local environment for survival. Chin J Popul Sci. (1994) 6:33–43. 12319205

[12] McDowell A Thompson S Stark M Ou ZQ Gould KS. Antioxidant activity of puha (*Sonchus oleraceus L.*) as assessed by the cellular antioxidant activity (CAA) assay. Phytother Res. (2011) 25:1876–82. 10.1002/ptr.3648 21928279

[13] Xia DZ Yu XF Zhu ZY Zou ZD. Antioxidant and antibacterial activity of six edible wild plants (*Sonchus spp.*) in China. Nat Prod Res. (2011) 25:1893–901. 10.1080/14786419.2010.534093 21793765

[14] Vilela FC Bitencourt AD Cabral LDM Franqui LS Soncini R Giusti-Paiva A. Anti-inflammatory and antipyretic effects of *Sonchus oleraceus* in rats. J Ethnopharmacol. (2010) 127:737–41. 10.1016/j.jep.2009.11.030 19962434

[15] Li XY Liu YH Wang B Chen CY Zhang HM Kang JX. Identification of a sustainable two-plant diet that effectively prevents age-related metabolic syndrome and extends lifespan in aged mice. J Nutr Biochem. (2018) 51:16–26. 10.1016/j.jnutbio.2017.09.003 29080417

[16] Vilela FC Padilha Mde M Alves-da-Silva G Soncini R Giusti-Paiva A. Antidepressant-like activity of *Sonchus oleraceus* in mouse models of immobility tests. J Med Food. (2010) 13:219–22. 10.1089/jmf.2008.0303 20136459

[17] Huyan T Li Q Wang YL Li J Zhang JY Liu YX. Anti-tumor effect of hot aqueous extracts from *Sonchus oleraceus (L.) L*. and *Juniperus sabina L*- two traditional medicinal plants in China. J Ethnopharmacol. (2016) 185:289–99. 10.1016/j.jep.2016.03.044 27001625

[18] Reagan-Shaw S Nihal M Ahmad N. Dose translation from animal to human studies revisited. FASEB J. (2008) 22:659–61. 10.1096/fj.07-9574LSF 17942826

[19] Bligh EG Dyer WJ. A rapid method of total lipid extraction and purification. Can J Biochem Physiol. (1959) 37:911–7. 10.1139/o59-099 13671378

[20] Taicher GZ Tinsley FC Reiderman A Heiman ML. Quantitative magnetic resonance (QMR) method for bone and whole-body-composition analysis. Anal Bioanal Chem. (2003) 377:990–1002. 10.1007/s00216-003-2224-3 13680051

[21] Unger RH Clark GO Scherer PE Orci L. Lipid homeostasis, lipotoxicity, and the metabolic syndrome. Biochim Biophys Acta. (2010) 1801:209–14. 10.1016/j.bbalip.2009.10.006 19948243

[22] Haider N Larose L. Harnessing adipogenesis to prevent obesity. Adipocyte. (2019) 8:98–104. 10.1080/21623945.2019.1583037 30848691 PMC6768234

[23] Jakab J Miskic B Miksic S Juranic B Cosic V Schwarz D. Adipogenesis as a potential anti-obesity target: a review of pharmacological treatment and natural products. Diabetes Metab Syndr Obes. (2021) 14:67–83. 10.2147/DMSO.S281186 33447066 PMC7802907

[24] Hardie DG. Sensing of energy and nutrients by AMP-activated protein kinase. Am J Clin Nutr. (2011) 93:891S−6S. 10.3945/ajcn.110.001925 21325438

[25] Chen H Liu X Chen H Cao J Zhang L Hu X. Role of SIRT1 and AMPK in mesenchymal stem cells differentiation. Ageing Res Rev. (2014) 13:55–64. 10.1016/j.arr.2013.12.002 24333965

[26] Fogarty S Hardie DG. Development of protein kinase activators: AMPK as a target in metabolic disorders and cancer. Biochim Biophys Acta. (2010) 1804:581–91. 10.1016/j.bbapap.2009.09.012 19778642

[27] Chen L Lin X Fan X Qian Y Lv Q Teng H. Sonchus oleraceus Linn extract enhanced glucose homeostasis through the AMPK/Akt/ GSK-3β signaling pathway in diabetic liver and HepG2 cell culture. Food Chem Toxicol. (2020) 136:111072. 10.1016/j.fct.2019.111072 31877369

[28] Chen L Teng H Cao H. Chlorogenic acid and caffeic acid from Sonchus oleraceus Linn synergistically attenuate insulin resistance and modulate glucose uptake in HepG2 cells. Food Chem Toxicol. (2019) 127:182–7. 10.1016/j.fct.2019.03.038 30914352

[29] Dagon Y Avraham Y Berry EM. AMPK activation regulates apoptosis, adipogenesis, and lipolysis by eIF2α in adipocytes. Biochem Biophys Res Commun. (2006) 340:43–7. 10.1016/j.bbrc.2005.11.159 16377306

[30] Sullivan JE Brocklehurst KJ Marley AE Carey F Carling D Beri RK. Inhibition of lipolysis and lipogenesis in isolated rat adipocytes with AICAR, a cell-permeable activator of AMP-activated protein kinase. FEBS Lett. (1994) 353:33–6. 10.1016/0014-5793(94)01006-4 7926017

[31] Dzamko N van Denderen BJ Hevener AL Jorgensen SB Honeyman J Galic S. AMPK beta1 deletion reduces appetite, preventing obesity, and hepatic insulin resistance. J Biol Chem. (2010) 285:115–22. 10.1074/jbc.M109.056762 19892703 PMC2804155

[32] Li Y Xu S Mihaylova MM Zheng B Hou X Jiang B. AMPK phosphorylates and inhibits SREBP activity to attenuate hepatic steatosis and atherosclerosis in diet-induced insulin-resistant mice. Cell Metab. (2011) 13:376–88. 10.1016/j.cmet.2011.03.009 21459323 PMC3086578

[33] Woods A Williams JR Muckett PJ Mayer FV Liljevald M Bohlooly YM. Liver-specific activation of AMPK prevents steatosis on a high-fructose diet. Cell Rep. (2017) 18:3043–51. 10.1016/j.celrep.2017.03.011 28355557 PMC5382239

[34] Bijland S Mancini SJ Salt IP. Role of AMP-activated protein kinase in adipose tissue metabolism and inflammation. Clin Sci. (2013) 124:491–507. 10.1042/CS20120536 23298225

[35] Lopez M Lage R Saha AK Perez-Tilve D Vazquez MJ Varela L. Hypothalamic fatty acid metabolism mediates the orexigenic action of ghrelin. Cell Metab. (2008) 7:389–99. 10.1016/j.cmet.2008.03.006 18460330

[36] Minokoshi Y Alquier T Furukawa N Kim YB Lee A Xue B. AMP-kinase regulates food intake by responding to hormonal and nutrient signals in the hypothalamus. Nature. (2004) 428:569–74. 10.1038/nature02440 15058305

[37] Villena JA Viollet B Andreelli F Kahn A Vaulont S Sul HS. Induced adiposity and adipocyte hypertrophy in mice lacking the AMP-activated protein kinase-alpha2 subunit. Diabetes. (2004) 53:2242–9. 10.2337/diabetes.53.9.2242 15331533

[38] Tang QQ Otto TC Lane MD. Mitotic clonal expansion: a synchronous process required for adipogenesis. Proc Natl Acad Sci U S A. (2003) 100:44–9. 10.1073/pnas.0137044100 12502791 PMC140878

[39] Tang QQ Otto TC Lane MD. CCAAT/enhancer-binding protein beta is required for mitotic clonal expansion during adipogenesis. Proc Natl Acad Sci U S A. (2003) 100:850–5. 10.1073/pnas.0337434100 12525691 PMC298690

[40] Chen Q Hao W Xiao C Wang R Xu X Lu H. SIRT6 is essential for adipocyte differentiation by regulating mitotic clonal expansion. Cell Rep. (2017) 18:3155–66. 10.1016/j.celrep.2017.03.006 28355567 PMC9396928

[41] Li Q Dong D-D Huang Q-P Li J Du Y-Y Li B. The anti-inflammatory effect of Sonchus oleraceus aqueous extract on lipopolysaccharide stimulated RAW 264.7 cells and mice. Pharm Biol. (2017) 55:799–809. 10.1080/13880209.2017.1280514 28112016 PMC6130567

[42] Kanda H Tateya S Tamori Y Kotani K Hiasa K-i Kitazawa R. MCP-1 contributes to macrophage infiltration into adipose tissue, insulin resistance, and hepatic steatosis in obesity. J Clin Investig. (2006) 116:1494–505. 10.1172/JCI26498 16691291 PMC1459069

[43] Ebron K Andersen CJ Aguilar D Blesso CN Barona J Dugan CE. A larger body mass index is associated with increased atherogenic dyslipidemia, insulin resistance, and low-grade inflammation in individuals with metabolic syndrome. Metab Syndr Relat Disord. (2015) 13:458–64. 10.1089/met.2015.0053 26431271

