# Supplementary material for: Lipid Extract From a Vegetable (Sonchus Oleraceus) Attenuates Adipogenesis and High Fat Diet-Induced Obesity Associated With AMPK Activation
Source: Front Nutr. 2021 Apr 6;8:624283. doi: 10.3389/fnut.2021.624283 (PMC8055827; doi:10.3389/fnut.2021.624283)
Supplement: Supplementary file 1 [file Data_Sheet_1.docx]

Supplementary Material


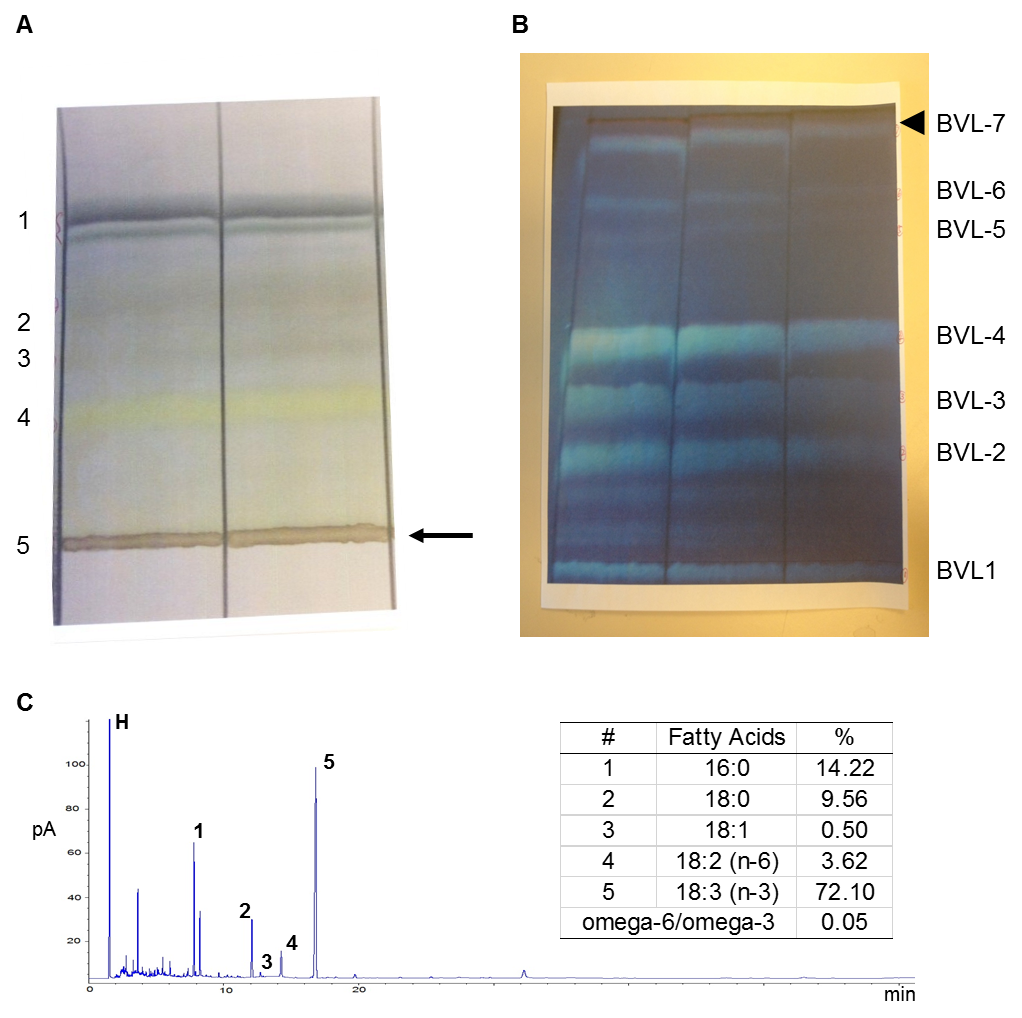


**Supplementary Figure 1.**

**(A)** The representative image showed that phospholipid-rich BVL (arrow) was obtained using TLC method. The mobile phase was consisted of Heptane: Diethyl Ether: Acetic Acid (60:40:3, v/v/v). **(B)** The representative image showed that BVL (arrowhead) was obtained using TLC method. The mobile phase was consisted of Chloroform: Methanol: Acetic acid (100:30:3, v/v/v). **(C)** The representative GC chromatogram showed the detection of fatty acids in BV-7. The table on the top-right corner showed the percentage of fatty acids to the corresponding standard and the ratio of omega-6 to omega-3 fatty acids.

**Supplementary Table 1. Parameters of body composition on mice after a 4-week intervention.**

| **Parameters** | **Control** | **BVL** | **p value** |
| --- | --- | --- | --- |
| *BW (g)* | 34.44±1.10 | 30.99±1.02 | 0.031 |
| ***Tissue Weight (g)*** | | | |
| *Ing* | 1.15±0.11 | 0.85±0.12 | 0.003 |
| *Epi* | 2.38±0.10 | 1.75±0.23 | 0.031 |
| *BAT* | 0.09±0.01 | 0.07±0.01 | 0.069 |
| *Liver* | 0.94±0.13 | 0.87±0.04 | 0.325 |
| *Mus* | 0.26±0.02 | 0.33±0.03 | 0.049 |
| ***Relative Percentage to BW (%)*** | | |  |
| *Ing* | 3.31±0.23 | 2.73±0.32 | 0.001 |
| *Epi* | 6.92±0.12 | 5.6±0.53 | 0.042 |
| *BAT* | 0.27±0.02 | 0.24±0.01 | 0.115 |
| *Liver* | 2.69±0.26 | 2.81±0.13 | 0.350 |
| *Mus* | 0.76±0.08 | 1.06±0.11 | 0.024 |

n=4 per group. The difference between two groups was considered significant when p < 0.05.

**
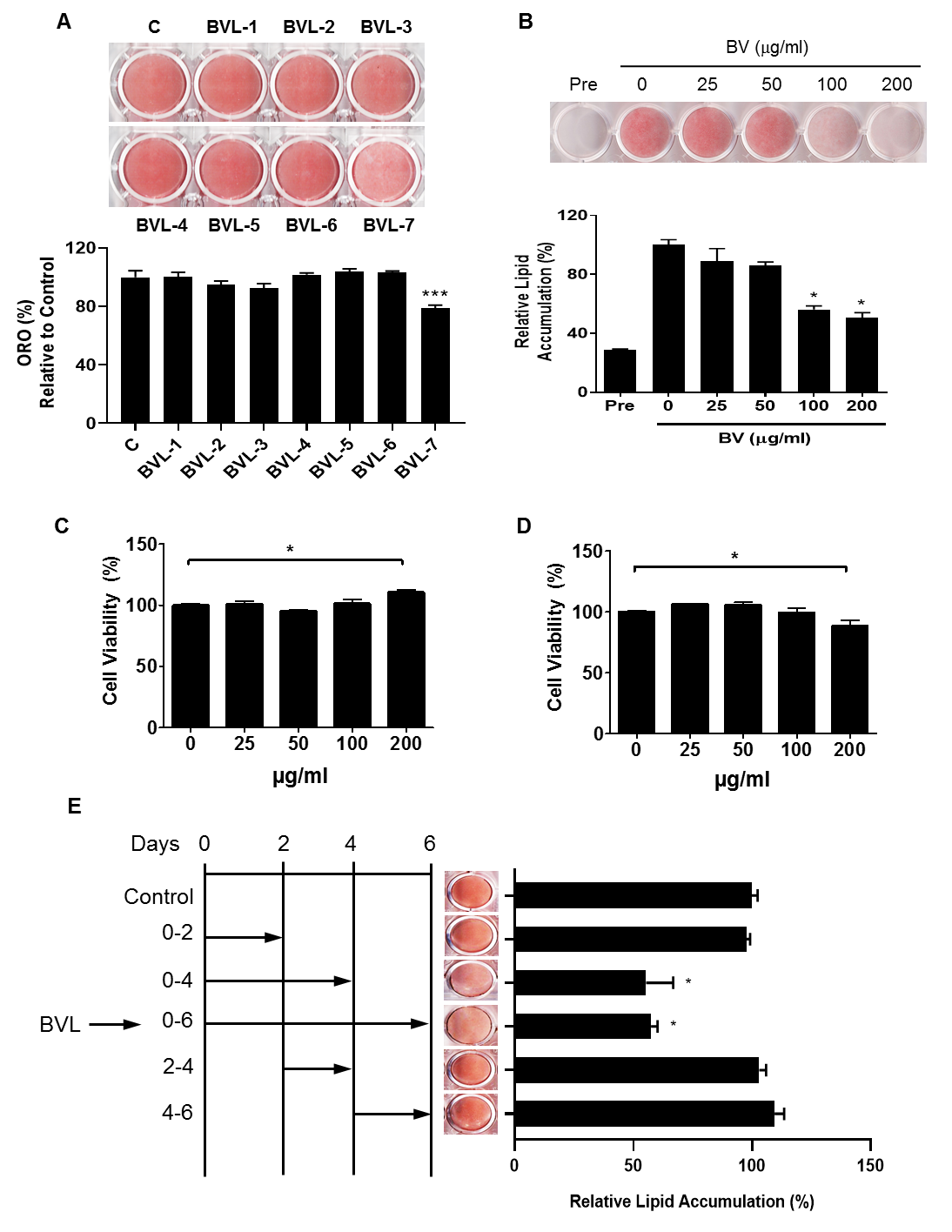
**

**Supplementary Figure 2. (A)** 3T3-L1 cells were differentiated with or without various fractions of BVL (BVL-1-BVL-7) at 75 µg/ml. ORO staining from adipocytes (D8) and quantitative analysis were shown. **(B)** 3T3-L1 cells were treated with indicated doses during the adipocyte differentiation. The representative ORO staining image and the quantitative analysis were shown. The cell viability in preadipocytes **(C)** and adipocytes **(D)** was detected using MTT assay. **(E)** 3T3-L1 preadipocytes were differentiated with or without BVL (100 µg/ml) at the indicated time. After 8 days of differentiation, ORO staining was performed, and the corresponding quantification was shown to determine the intracellular lipid content. The values were presented using mean ± SEM and each experiment was repeated at least 3 times. * p<0.05.


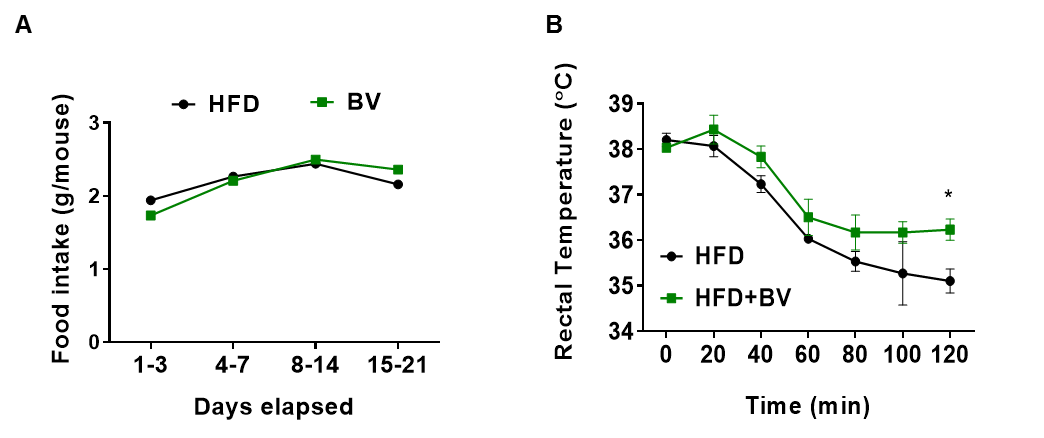


**Supplementary Figure 3. (A)** Food intake. **(B)** Rectal core body temperature of HFD fed mice and HFD+BV fed mice was measured at 4°C. * p<0.05 (n=4).
